# Prognostic Relevance of Cytochrome c Oxidase in Primary Glioblastoma Multiforme

**DOI:** 10.1371/journal.pone.0061035

**Published:** 2013-04-10

**Authors:** Corinne E. Griguer, Alan B. Cantor, Hassan M. Fathallah-Shaykh, G. Yancey Gillespie, Amber S. Gordon, James M. Markert, Ivan Radovanovic, Virginie Clement-Schatlo, Chevis N. Shannon, Claudia R. Oliva

**Affiliations:** 1 Division of Neurosurgery, Department of Surgery, University of Alabama at Birmingham, Birmingham, Alabama, United States of America; 2 Departments of Neurology and Mathematics, University of Alabama at Birmingham, Birmingham, Alabama, United States of America; 3 Department of Medicine, Division of Preventative Medicine, University of Alabama at Birmingham, Alabama, United States of America; 4 Division of Neurosurgery, Geneva University Hospitals and University of Geneva, Geneva, Switzerland; University of Portsmouth, School of Pharmacy & Biomedical Sciences, United Kingdom

## Abstract

Patients with primary glioblastoma multiforme (GBM) have one of the lowest overall survival rates among cancer patients, and reliable biomarkers are necessary to predict patient outcome. Cytochrome c oxidase (CcO) promotes the switch from glycolytic to OXPHOS metabolism, and increased CcO activity in tumors has been associated with tumor progression after chemotherapy failure. Thus, we investigated the relationship between tumor CcO activity and the survival of patients diagnosed with primary GBM. A total of 84 patients with grade IV glioma were evaluated in this retrospective cohort study. Cumulative survival was calculated by the Kaplan-Meier method and analyzed by the log-rank test, and univariate and multivariate analyses were performed with the Cox regression model. Mitochondrial CcO activity was determined by spectrophotometrically measuring the oxidation of cytochrome *c.* High CcO activity was detected in a subset of glioma tumors (∼30%), and was an independent prognostic factor for shorter progression-free survival and overall survival [P = 0.0087 by the log-rank test, hazard ratio = 3.57 for progression-free survival; P<0.001 by the log-rank test, hazard ratio = 10.75 for overall survival]. The median survival time for patients with low tumor CcO activity was 14.3 months, compared with 6.3 months for patients with high tumor CcO activity. High CcO activity occurs in a significant subset of high-grade glioma patients and is an independent predictor of poor outcome. Thus, CcO activity may serve as a useful molecular marker for the categorization and targeted therapy of GBMs.

## Introduction

Glioblastoma multiforme (GBM) is the most frequent form of high-grade malignant brain tumor. Since the study by Stupp et al. in 2005, the standard of care treatment for patients with primary GBM has included a regimen of concomitant and adjuvant chemotherapy with temozolomide (TMZ) [1], [2]. However, the life expectancy of patients with GBM remains short, with a median survival of 14 months [3]. Considerable efforts have been made to define molecular signatures in GBMs that can be used as prognostic or predictive markers. Methylation of the *O^6^*-methylguanine (*O^6^*-meG) DNA methyltransferase (MGMT) promoter is a reliable predictor of clinical response to TMZ. High levels of MGMT promoter methylation correlate with a favorable response to TMZ [1]. Recently, mutations in the isocitrate dehydrogenase-1 (IDH1) gene have been identified as a major prognostic marker for overall and progression-free survival in secondary GBMs [4], [5].

Increased levels of cytochrome c oxidase (CcO, complex IV; EC 1.9.3.1) activity have been associated with the acquisition of chemoresistance to TMZ in malignant gliomas [6], [7]. CcO is the terminal enzyme of the mitochondrial respiratory chain (electron transport chain, ETC) that catalyzes the transfer of electrons from cytochrome *c* to oxygen (O_2_). CcO is a complex enzyme consisting of 13 subunits, three of which are encoded by the mitochondrial DNA (mtDNA) and perform the catalytic function, and 10 of which are nuclear-encoded and provide the regulatory function [8], [9]. Increased CcO activity augments the electron flux capacity of the ETC, leading to more efficient mitochondrial coupling and reduced production of reactive oxygen species (ROS) [6], [7], [10], [11]. These alterations are likely to facilitate adaptive chemoresistance through the suppression of apoptotic signaling [12]. We have recently demonstrated that inhibiting CcO activity reverses chemoresistance to TMZ [6], [7], supporting a close correlation between acquired chemoresistance and changes in cellular metabolic machinery at the level of the mitochondrion. Thus, we propose that regulation of bioenergetics in cancer cells plays an essential role in tumor progression. Furthermore, we speculate that chemoresistant cells result primarily from the clonal selection of TMZ-resistant cells, present in the original polyclonal population, in which ROS production was suppressed before the TMZ challenge. In this retrospective cohort study, we investigated whether primary tumor CcO activity is associated with overall and progression-free survival of glioma patients.

## Materials and Methods

### Ethics Statement

The protocol for this study was approved by the Institutional Review Board for Human Use at the University of Alabama at Birmingham (UAB) (IRB #X050415007 and X110418007). All patients (training and validation sets) provided written informed consent to the surgical procedures and gave permission for the use of resected tissue specimens.

### Acquisition of Tissue Specimens

90 samples were obtained but only 58 met requirement for a diagnosis of primary untreated GBM and containing a sufficient amount of tissue of appropriate quality to obtain both CcO and CS activity profiles. Frozen glioma tissue specimens (58 samples, training set) and normal brain tissue specimens from epilepsy patient were obtained from the collection of clinical specimens in the UAB Brain Tumor Tissue Bank from patients who underwent surgical treatment at the UAB Hospital between January 2001 and November 2011, spanning époques that included treatment without and then with temozolomide therapy. None of these patients received chemotherapy or radiotherapy before the surgery. Additionally, we conducted a retrospective evaluation of 26 frozen GBM samples (validation set) obtained from patients who underwent surgical treatment between March 2005 and September 2011 at the University of Geneva, Geneva, Switzerland. None of these patients received chemotherapy or radiotherapy before surgery.

### Mitochondrial Isolation

Isolation of mitochondria from GBM tumors was performed as previously described [13]. Briefly, each piece of tumor was weighed, minced, and suspended with ice-cold isolation buffer (250 mM sucrose, 10 mM Tris-HCl, 0.5 mM EDTA; pH 7.4), then manually homogenized. The homogenate was centrifuged for 5 minutes at 1000×*g*, and the pellets (nuclear enriched fractions) were frozen prior to DNA isolation. The supernatants were centrifuged for 10 minutes at 12,500×*g*, and the pellets were frozen prior to CcO activity determination.

### Enzymatic Activities

Spectrophotometric determination of citrate synthase (CS; EC 4.1.3.7) and CcO activity levels was performed as we previously described [6], [7]. Briefly, CS activity was measured at 415 nm, in potassium phosphate buffer, pH 7.2, with the addition of 2.5 mM dithionitrobenzoic acid, 2.5 mM acetyl-CoA and 10 mM oxaloacetate. The increase in absorbance was used to calculate CS enzyme activity. CcO activity was measured in potassium phosphate buffer, pH 7.2, with the addition of 10 µM reduced Cyt *c*. The oxidation of Cyt *c* was measured as the decrease in absorbance at 550 nm and was used to calculate CcO enzyme activity. CcO activity was expressed as micromoles of cytochrome c oxidized per second per mg protein. The activity of CS, a Krebs cycle enzyme, remains stable in isolated mitochondria. Therefore, CS activity was used to normalize CcO activity [8], [14], [15].

### DNA Isolation

Nuclear enriched fractions isolated from tumor tissues were subjected to digestion with 1% sodium dodecyl sulfate (SDS), 50 mM EDTA in 20mM Tris-HCl (pH 8), and proteinase K at 37°C for 20 hours. DNA was then isolated by standard Trizol protocol and stored at −80°C until use.

### Methylation Specific PCR (MSP)

DNA methylation patterns of the MGMT gene were determined by chemical modification of unmethylated cytosines to uracil and subsequent PCR using primers specific for either methylated or modified unmethylated DNA, according to Esteller et al. [16] DNA was treated with sodium bisulfite as previously described. [17] Briefly, 4 µg of genomic DNA was incubated with 0.3 M NaOH at 50°C for 20 minutes to denature the DNA. The mixture was then incubated for 20 hours at 50°C in 500 µl of a freshly prepared solution containing 3 M sodium bisulfite and 10 mM hydroquinone. DNA was subsequently purified with a Wizard DNA Clean-Up System, following the instructions of the manufacturer, then resuspended in 100 µl of deionized H2O and stored at −80°C until use. Primer sequences for the unmethylated MGMT were: 5′-TTTGTGTTTTGATGTTTGTAGGTTTTTGT-3′ (forward primer) and 5′-AACTCCACACTCTTCCAAAAACAAAACA-3′ (reverse primer). Primer sequences for the methylated MGMT were: 5′-TTTCGACGTTCGTAGGTTTTCGC-3′ (forward primer) and 5′-GCACTCTTCCGAAAACGAAACG-3′ (reverse primer) [16].

### Statistical Analysis

Progression-free survival was defined as the time from initial surgery of the primary tumor to tumor recurrence, as detected by clinical and radiographic evidence of progression. Overall survival was defined as the time from the date of pathologic diagnosis to death due to any cause. Times for those not experiencing the endpoint were censored as of the last date known to be event free. Log-rank test analysis was performed to determine the cutoff score for high CcO activity. A CcO/CS ratio of 4 gave the smaller p value, and was selected as the cutoff value. Tumors with scores below or equal to the cutoff value were categorized as having low CcO activity, while tumors with scores above the value were categorized as having high CcO activity.

The Kaplan–Meier method was used to estimate the survival rates, and the log-rank test was used to compare rates between cohorts. Patients who remained alive at last follow-up were considered censored events in our analysis. Multivariate survival analysis was performed using the Cox regression model. The significance threshold was set at a two-sided P value of <0.05 for all analyses. Data are shown as the means ± S.E.

## Results

**Figure 1:**
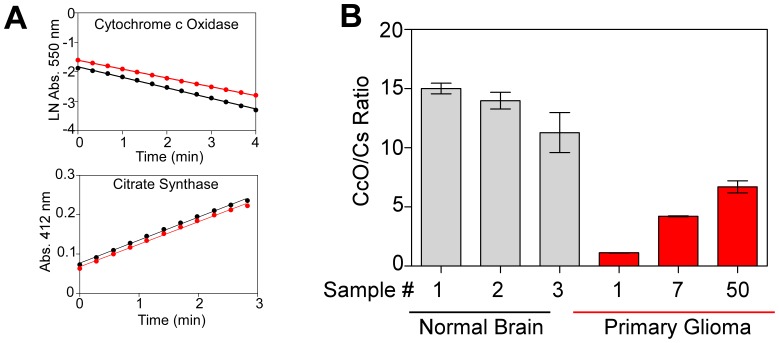
Determination of CcO Activity Levels. **Panel A** shows the representative time course curves of CcO activity (top) and CS activity (bottom). CcO activity was determined by spectrophotometrically measuring the oxidation of cytochrome *c*, as indicated by the decrease of absorbance at 550 nm. CS activity was determined by measuring spectrophotometric thionitrobenzoic acid (TNB) production, as indicated by the increase of absorbance at 412 nm. For each tissue sample, activities were measured at least twice at protein concentrations that ensured the linearity of the reaction. CcO-specific and CS-specific activities were calculated using the slopes of the curves, and activities were expressed as nanomoles of cytochrome *c* oxidized per minute per milligram of protein. Red and black lines denote duplicate determinations of a representative tissue. **Panel B** shows representative CcO/CS ratios from normal brain (epilepsy patients, gray bars) and from primary glioma tissue samples (red bars). Bars represent the average of at least two independent determinations ± SEM.

**Figure 2:**
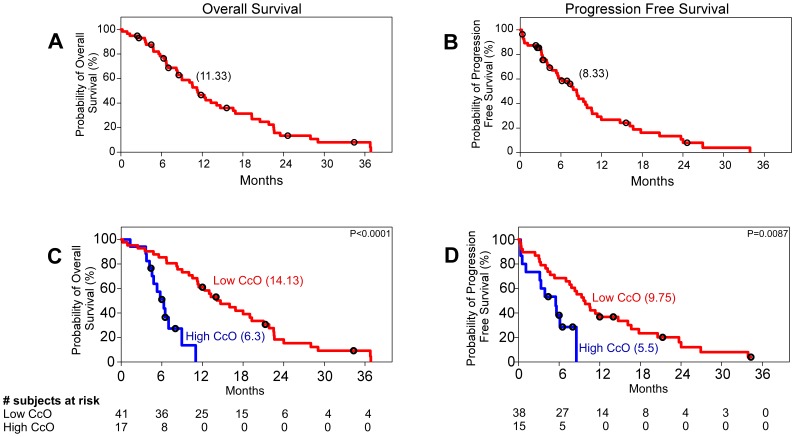
Kaplan–Meier Estimates of Overall Survival and Progression Free Survival. **Panel A** shows the probability of overall survival and **Panel B** shows the probability of progression-free survival for the 58 patients with grade IV primary glioblastoma in the training set. **Panel C** shows the difference in the probability of overall survival for patients with high and low tumor CcO activity (P<0.0001 by the log-rank test; hazard ratio for death in patients with high tumor CcO activity, 10.75; 95% CI, 3.79 to 30.51). **Panel D** shows the difference in the probability of progression-free survival for patients with high and low tumor CcO activity (P = 0.0087 by the log-rank test; hazard ratio for death in patients with high tumor CcO activity, 3.57; 95% CI, 1.38 to 9.22). Black circles denote censored points, and the numbers between brackets indicate the median survival for each group. CcO denotes cytochrome c oxidase.

We first determined the levels of CcO and CS activity in mitochondria isolated from 58 primary glioma tissues (training samples, Birmingham cohort) and 12 tissue samples from normal brain (epilepsy patients). CS activity was used to normalize CcO activity in each sample [8], [14], [15]. Representative time courses of CcO and CS activities (duplicate determinations), as well as representative CcO/CS ratios for normal brain tissue and primary gliomas, are presented in Figure 1. The mean CcO/CS ratio for normal brain was 14.33±3.77 (n = 12), whereas the mean CcO/CS value for the Birmingham glioma cohort was 4.48±0.59 (minimum, 0.376; maximum, 21.63, n = 58, p<0.0001). For the entire training population, median overall survival was 11.33 months, and progression-free survival was 8.33 months (Figure 2A and B). However, when patients were stratified by CcO activity, there was a significant difference in overall survival and progression-free survival between patients whose tumors had high (CcO/CS>4) activity or low CcO (CcO/CS≤4) activity. High CcO activity was detected in 17 patients (30%) from the Birmingham glioma cohort and was associated with lower progression-free survival and overall survival. The median overall survival among patients with low tumor CcO activity was 14.13 months (95% confidence interval [CI], 12.37 to 18.19), as compared with 6.3 months (95% CI, 4.60 to 6.92) among patients with high tumor CcO activity (P<0.0001 by the log-rank test) (Figure 2C). The hazard ratio for death was 10.75 (95% CI, 3.79 to 30.51) among those with high tumor CcO activity, a result that corresponds to a 10-fold increase in the risk of death in this subgroup. High tumor CcO activity was also strongly associated with shorter progression-free survival. The median progression-free survival among patients with low tumor CcO activity was 9.75 months (95% CI, 8.47 to 14.30), as compared with 5.5 months (95% CI, 2.72 to 5.74) among patients with high tumor CcO activity (P = 0.0087 by the log-rank test) (Figure 2D). The hazard ratio was 3.57 (95% CI, 1.38 to 9.22) among patients with high tumor CcO activity.

**Figure 3:**
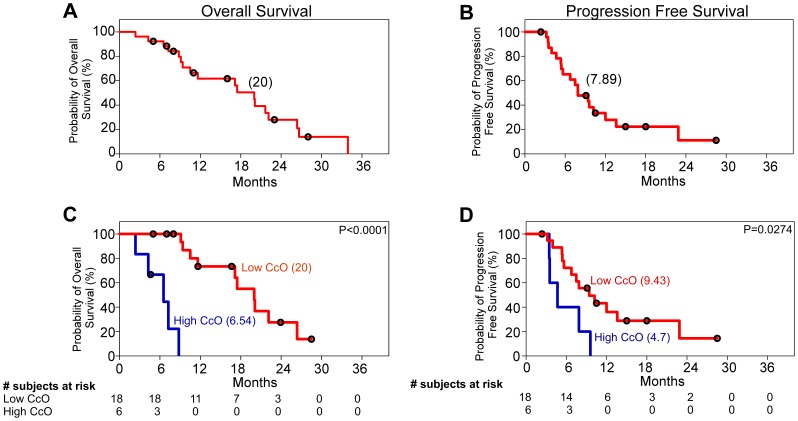
Kaplan–Meier Estimates of Overall Survival and Progression Free Survival in an Independent Validation Set. A total of 27 primary glioma tissue samples from the University of Geneva, Switzerland, were analyzed in the validation set. **Panel A** shows the probability of overall survival and **Panel B** shows the probability of progression-free survival for these patients. **Panel C** shows the difference in the probability of overall survival for patients with high and low tumor CcO activity (a P<0.0001 by the log-rank test; hazard ratio for death in patients with high tumor CcO activity, 17; 95% CI, 16.69 to 18.07). **Panel D** shows the difference in the probability of progression-free survival for patients with high and low tumor CcO activity (hazard ratio for death in patients with high CcO activity, 5.31; 95% CI, 1.2 to 23.58). Black circles denote censored points, and the numbers between brackets indicate the median survival for each group. CcO denotes cytochrome c oxidase.

To confirm the prognostic value of tumor CcO activity, we performed a blinded validation study in an independent series of 26 patients with primary GBM whose tissues were collected at the University of Geneva, Switzerland. The median overall survival and progression-free survival of the entire validation set were 20.00 and 7.89 months, respectively (Figure 3A and B). In this validation series, high tumor CcO activity was identified in 6 patients (25%) and was also strongly associated with shorter overall survival. The median overall survival among patients with low tumor CcO activity was 20 months (95% CI, 19.09 to 22.16), as compared with 6.54 months (95% CI, 3.2 to 8.08) among patients with high tumor CcO activity (P<0.0001 by the log-rank test) (Figure 3C). High tumor CcO activity was also strongly associated with shorter progression-free survival. The median progression-free survival among patients with low tumor CcO activity was 9.43 months (95% CI, 8.47 to 14.30), as compared with 4.7 months (95% CI, 2.72 to 5.74) among patients with high tumor CcO activity (P = 0.0274 by the log-rank test) (Figure 3D).

**Figure 4:**
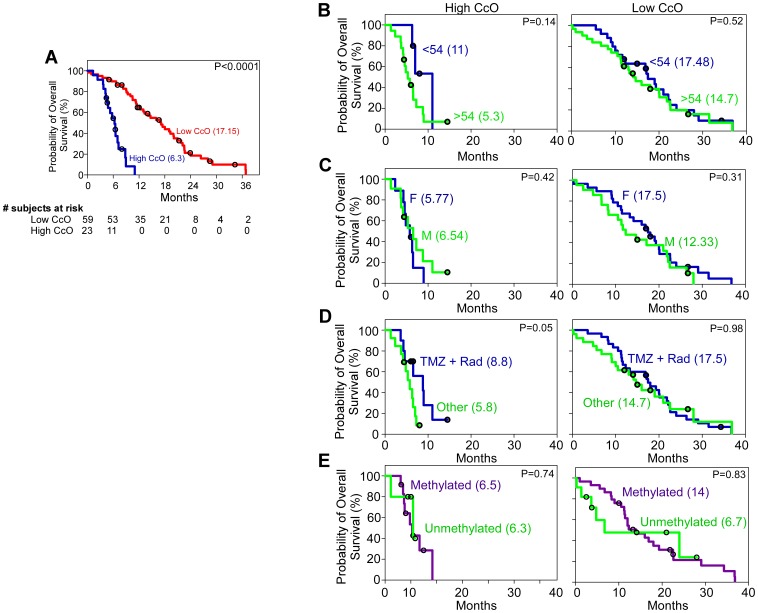
Multivariate Analysis of Survival for the Cohorts with High and Low CcO Activity. **Panel A** shows the Kaplan-Meier estimate of the probability of overall survival, according to CcO activity, in all 84 patients combined from the training and validation tissue cohorts (P<0.0001 by the log-rank test; hazard ratio for death in patients with high CcO activity, 24.20; 95% CI, 9.12 to 34.20). Red denotes tumors with low CcO activity and blue denotes tumors with high CcO activity. **Panel B, C, D, and E** show the probability of survival in patients with high (left) and low (right) CcO activity with respect to age (Panel B; hazard ratio, 0.24; P = 0.14), gender (Panel C; hazard ratio, 0.58; P = 0.42), treatment administered (Panel D; hazard ratio, 0.39; P = 0.05) and MGMT promoter methylation status (Panel E; hazard ratio, 0.9; P = 0.83). Black circles denote censored points, and the numbers between brackets indicate the median survival for each group. CcO denotes cytochrome c oxidase, F denotes female, M denotes male, TMZ denotes temozolomide, and Rad denotes radiotherapy.

We also tested for interactions between tumor CcO activity and single clinical prognosis/predictive parameters regarding overall survival, combining patients from both populations (Birmingham and Geneva), but did not find any relevant interaction (Figure 4). The median overall survival of the combined populations of 82 patients was 13.27 months (95% CI, 10.81 to 14.55). However, when the patients were stratified by tumor CcO activity, there was a significant difference in overall survival. High tumor CcO activity was detected in 23 patients (28%) of the combined cohorts and was associated with lower overall survival. The median overall survival among patients with low tumor CcO activity was 17.15 months (95% CI, 13.16 to 17.61), as compared with 6.3 months (95% CI, 4.77 to 6.69) among patients with high tumor CcO activity (P<0.0001 by the log-rank test) (Figure 4A). The hazard ratio for death was 24.20 (95% CI, 9.12 to 34.20) among patients with high tumor CcO activity, a result that corresponds to a 25-fold increase in the risk of death in this subgroup.

Finally, we established multivariate survival models for overall survival including the following prognostic factors: age at diagnosis, gender, *MGMT* promoter methylation status, and therapy. To analyze the effect of therapy, the patients were divided into two populations. One population included all patients receiving radiotherapy plus TMZ as the first line of treatment, and the second population included patients receiving either radiotherapy alone, TMZ alone, any other therapeutic option or no treatment. The models were designed to take each of the factors into account without considering interaction terms. As shown in Figure 4, we found that high tumor CcO activity was not associated with age (hazard ratio, 0.24; P = 0.14), gender (hazard ratio, 0.58; P = 0.42), the treatment administered (hazard ratio, 0.39; P = 0.05), or *MGMT* promoter methylation status (hazard ratio, 1.28; P = 0.74). Similarly, low tumor CcO activity was not associated with age (hazard ratio, 0.57; P = 0.52), gender (hazard ratio, 0.56; P = 0.31), the treatment administered (hazard ratio, 0.84; P = 0.98), or *MGMT* promoter methylation status (hazard ratio, 0.9; P = 0.83).

## Discussion

CcO is an important mitochondrial multi-protein complex with two main substrates– oxygen and cytochrome c. The function of CcO as an electron carrier is well known; however, its utility as a prognostic factor has never been reported. Previous studies demonstrated that CcO is critically involved in establishing resistance to apoptosis in cervical cancer cells [10], [11] and gliomas [6], [7]. Thus, the present study was designed to determine whether the level of tumor CcO activity is associated with clinical outcomes in primary GBM patients. Our results demonstrate that CcO activity is linked to both overall survival and progression-free survival in patients with newly diagnosed GBM. Specifically, high tumor CcO activity is significantly associated with poor prognosis, whereas low tumor CcO activity is associated with better outcomes.

CcO status appears to be a robust prognostic factor when compared with the most reliable predictor of tumor susceptibility, *MGMT* promoter methylation [1]. A significant fraction of the Birmingham training set was obtained prior to standard use of temozolomide, resulting in an overall decreased median survival for this cohort when compared to more modern cohorts. Nonetheless, the finding of high vs low CcO activity predicting overall and progression free survival holds and was actually established in a mixed group, indicating that this finding holds true even within the range of chemotherapeutic regimens utilized for these patients. The mechanism(s) underlying this phenomenon is unknown at this point. One possible explanation is that high CcO activity may confer a selective advantage during the progression of the tumor, in particular under oxidative stress insults, nutrient deprivation, and/or hypoxic conditions [10], [11]. Indeed, it has been shown that neurons in the corpus callosum have low CcO activity and are highly susceptible to injuries and oxidative stress when compared with neurons in the cerebral cortex that have higher CcO activity [18]. A higher respiratory capacity in a subpopulation of glioma cells may also inhibit cancer cell apoptosis by preventing the early release of cytochrome *c* into the cytosol [19], [20]. Considering that the level of cytochrome *c* rarely exceeds the concentration required for CcO activity [21]–[23], an increase in CcO activity will likely decrease the pool of cytochrome *c* available to initiate apoptosis, rendering cells more resistant to therapy.

Patients with primary GBM have one of the lowest overall survival rates among cancer patients, in part due to the molecular heterogeneity of GBMs. This poses many challenges for the development of novel therapies. In this study, we were able to identify a subset of patients (25–30% of the entire population) with primary GBM with an extremely low overall survival. Interestingly, the median overall survival rate in this subgroup was the same in both analyzed populations (6.3 and 6.4 months for Birmingham and Geneva, respectively). We speculate that tumors in this population may represent a novel primary GBM subtype characterized by less intratumoral heterogeneity, OXPHOS metabolism, and resistance to stress insults including radio and chemotherapies. However, further studies are needed to characterize the molecular mechanism(s) underlying the more aggressive phenotype of this population.

This study focused specifically on primary GBM; however, whether or not these findings are applicable to other gliomas remains to be demonstrated. Our previous analysis of CcO activity in GBM biopsies from patients subjected to TMZ-radiotherapy [6], [7] demonstrated that the CcO activity in recurrent tumors is significantly greater than the activity in primary tumors, suggesting that during tumor regrowth, a switch from glycolytic (low CcO activity) to OXPHOS metabolism (high CcO activity) may occur.

Overall, patients with primary GBM and high tumor CcO activity display the worst clinical outcomes, whereas patients with low tumor CcO activity have better outcomes. Despite the strong evidence across discovery and validation sets, prospective validation of the relationship between CcO activity levels and patient survival is needed. However, CcO status will be useful as a robust prognostic tool and clinical trial selection criteria, and represents an important step toward achieving personalized therapy for GBM patients.
